# Experimental data-set for prediction of tool wear during turning of Al-1061 alloy by high speed steel cutting tools

**DOI:** 10.1016/j.dib.2018.04.003

**Published:** 2018-04-12

**Authors:** I.P. Okokpujie, O.S. Ohunakin, C.A. Bolu, K.O. Okokpujie

**Affiliations:** a Department of Mechanical Engineering, Covenant University, Ota, Ogun State, Nigeria; b Department of Electrical and Information Engineering, Covenant University, Ota, Ogun State, Nigeria

**Keywords:** Tool wear, Turn operation, Aluminium 1061 alloy, High speed steel

## Abstract

In this investigation, the dataset presented will give important information to understand the area of cutting tool wear during turning operations, tool nature is the most difficult tasks in manufacturing process, particularly in the locomotive industry. With the view to optimize the cutting parameters, the tests were carried out to investigate tool wear on high speed steel (HSS) during turning operation of aluminium 1061 alloy and to developed mathematical models using least squares method. The cutting parameters chosen for this investigation are cutting speed, feed rate, and radial depth of cut were used as input parameters in order to predict tool wear. The experiment was designed by using full factorial 3^3^ in which 27 samples were run in a Fanuc 0i TC CNC lathe. After each test, scanning electron microscope (SEM) is used to measure the cutting tool in other to determine the tool wear.

**Specifications Table**

**Table:** 

Subject area	*Mechanical, Industrial and Production Engineering*
More specific subject area	*Design Engineering*
Type of data	*Table, image*
How data was acquired	*The high speed steel cutting tools was measured using JSM-6700F scanning electron microscope (SEM) after each experiment.*
Data format	*Raw, Analysed*
Experimental factors	*The machining parameters such as cutting speed of 150, 200 and 250* *m/min, feed rate of 50, 100, and 150* *mm/min, and radial depth of cut of 0.5, 1, and 1.5* *mm was used as input variables during the turning operation of the Al-1061 in order to predict tool wear, and to determine the effects of the cutting parameters on the tool wear.*
Experimental features	*The work piece material used for the investigation is an aluminium 1061 alloy round steel bars with dimensions of length of 380* *mm and diameter of 38* *mm, obtained from Eddyson Aluminium and Steel Ltd., at Line 2, No. 10 Owode-Onirin Aluminium International Markets Lagos. The turning process was orthogonal. The experiments were performed by turning Al-1061 material using high speed steel tools on FANUC 0i TC CNC lathe with GA 2000, Spindle Motor 15 Kw Spindle diameter 51* *mm the experimental analysis was done at the Prototype Engineering Development Institute and the machining workshop at Covenant University Ota Ogun State, Nigeria.*
Data source location	*The Prototype Engineering Development Institute, Located at Ilesha, Oshogbo, Osun State and Department of Mechanical Engineering, Covenant University, Ota Ogun State, Nigeria.*
Data accessibility	*Data are available within this article*

**Value of the data**

• The experimental data will show author and manufacturers in the field of Mechanical engineering design the relationship and effect between the cutting tool, work-piece and the machining parameters.
• The data is useful in providing the optimization parameters for the machining operations.
• The data can be used to examine the interactions of the machining parameters for example (cutting speed and radial depth of cut or feed rate and cutting speed) as it influences the nature of tool wear produced.

## 1 Data

Tool wear, which gives rise to tool replacement, is most significant economic consequence to take into consideration during turning operation [1], [2]. Wear is unwanted deterioration of an element by the elimination of some quantity from the surface of the work piece. It occurs by the removal of chips from the work piece [3]. Tool deformation being a tribological phenomenon forms with frequent machining and then lead to increase in surface irregularity [3]. Process parameters such as feed rate, depth of cut and cutting speed, do influence the product quality and production costs. One of the main reasons in the optimization of a turning process is reduce vibration and heat generated which will lead to increase in tool wear [4]. Thus, it is important to use optimization technique to resolve optimal levels of these parameters so as to reduce idle time, the production costs and to attain the desired product quality simultaneously [5]. The cost of machining is strongly related to the tool wear. High Speed Steel tool (HSS) is a cutting tool that contains high carbon and large quantity of tungsten [6], [7], [8].

## 2 Experimental design, materials and methods

The work piece material used for the experiment is aluminium 1061 alloy round steel bars with dimensions of length of 380 mm and diameter of 38 mm, the turning process was orthogonal. Experiments were performed by machining Al-1061 material using high speed steel tools on Fanuc 0i TC CNC lathe, the experimental investigation was done at the Engineering Development Institute Machining Workshop. The boundary condition of the work piece is that both chuck ends is clamped (c-c). After each turning operation the SEM was used to measured cutting tool. The result was used to develop a model for the prediction of the cutting tool wear. In order to design the experimental plan, for the turning operation and for the development of the model, least squares method was employed. Experiment with three factors and three levels were used. According to these 3^3^ designs which give a total of 27 experimental run, the parameters for the experiments were selected from analytical results for both stable and unstable zones.

## 3 Mathematical models

The relationship between the tool wear and cutting parameters is shown in Eq. (1)

(1) $${\mathrm{Tw}}_{\max}=K{V.}^{x}{f.}^{y}$$

where *K* is constant, and *x, y,* and *r* are the power equations. Eq. (2) can be represented in mathematical form as follows: [9]

(2) $$\log{\mathrm{Tw}}_{\max}=\log\;K+x.\log\;V+y.\log\;f+z.\log\;r+\mathrm{zr}.\log\;r$$

The constant and exponents *K*, *x*, *y*, *z*, can be determined by least squares method. The introduction of a replacement gets the following expression:

(3) $$\begin{array}{c} Y=\;\log\;Tw_{max},\;\beta_{0}\;=\;\log\;K,\;x_{1}\;=\;\log\;V,\;x_{2}=\;\log\;f,\;x_{3}\;=\;\log\;r,\;x\;=\beta_{1}\\ y\;=\beta_{2},\;z=\beta_{3}\\ \mathrm{Therefore},\;10_{0}^{\beta }\;=K \end{array}$$

Linear model developed from the equation can be represented as follows:

(4) $$Y={\;\beta }_{0\;}+\;\beta_{1}x_{2}\;+\;\beta_{2}x_{2}\;+\;\beta_{3}x_{3}\;+\;e$$

where *x*_1_, *x*_2_, *x*_3_, are base-10 logarithmic transformation of factors: spindle speed, feed rate, axial depth of cut and radial depth of cut and *β* values are the estimates of corresponding parameters.

From Eq. (4), by minimizing the sum of the squares of the residual,

We have

(5) $$S_{r}=\sum_{i=1}^{n}{\left[Y_{i}-(\beta_{0\;}+\;\beta_{1}x_{1\;}+\;\beta_{2}x_{2}+\;\beta_{3}x_{3}\right]}^{2\;}$$

Solving the minimization, the resulting equations are as follows

(6a) $${n\beta }_{0}+\beta_{1}{\sum x}_{1}+\beta_{2}\sum x_{2}+\beta_{3}\sum x_{3}=\sum Y_{1}$$

(6b) $$\beta_{0}\sum x_{1}+\beta_{1}\sum x_{1}^{2}+\beta_{2}\sum x_{1}x_{2}+\beta_{3}\sum x_{1}x_{3}=\sum x_{1}Y_{i}$$

(6c) $$\beta_{0}\sum x_{2}+\beta_{1}\sum x_{1}x_{2}+\beta_{2}\sum x_{2}^{2}+\beta_{3}\sum x_{2}x_{3}=\sum x_{2}Y_{i}$$

(6d) $$\beta_{0}\sum x_{3}+\beta_{1}\sum x_{1}x_{3}+\beta_{2}\sum x_{2}x_{3}+\beta_{3}\sum x_{3}^{2}=\sum x_{3}Y_{i}$$

Since the tool wear from the experiment has been established, the analysis for the multiple regressions using equations above are done to obtain regression coefficient and the sum values calculated for $x_{i}$ with the following results:

$$\sum x_{1}=61.87555,\;\sum x_{2}=52.87555,\;\sum x_{3}=-1.12445,\sum Y_{i}=-13.2403,\sum x_{1}x_{2}=121.1742,\;\sum x_{1}x_{3}=-2.57688,\;\sum x_{1}Y_{i}=-30.2661,\;\sum x_{1}^{2}=142.0221,\;\sum x_{2}x_{3}=-2.20207,\;\sum x_{3}^{2}=1.094645,\;\sum x_{3}Y_{i}=0.553191,\;\sum x_{2}^{2}=104.5969,\;\sum x_{2}Y_{i}=-25.8868$$

Substituting all the sums values, into the simultaneous equation of linear system, as follows:

$${27\beta }_{0}\;+\;{61.87555\beta }_{1}\;+\;{52.87555\beta }_{2}{\;-\;1.12445\beta }_{3}=-13.2403{61.87555\beta }_{0}\;+\;{142.0221\beta }_{1}+{121.1742\beta }_{2}\;{-\;2.57688\beta }_{3}=-30.2661{52.87555\beta }_{0}\;+\;{121.1742\beta }_{1}\;+\;{104.5969\beta }_{2}\;{-\;2.20207\beta }_{3}=-25.8868\;{-\;1.12445\beta }_{0}{\;-\;2.57688\beta }_{1}{\;–\;2.20207\beta }_{2}{+\;1.094645\beta }_{3}=0.553191$$

Transform above equations into matrix form

$$\left[\begin{array}{ccc} 27 & 61.8755 & 52.8755\\ 61.8755 & 142.0221 & 121.1742\\ 52.8755 & 121.1742 & 104.5969\\ -1.12445 & -2.57688 & -2.20207 \end{array}\begin{array}{c} -1.12445\\ -2.57688\\ -2.20207\\ 1.094645 \end{array}\right]\left\{\begin{array}{c} \beta_{0}\\ \beta_{1}\\ \beta_{2}\\ \beta_{3} \end{array}\right\}=\left\{\begin{array}{c} -13.2403\\ -30.2661\\ -25.8868\\ 0.553191 \end{array}\right\}$$

Solving the above equations to get the coefficient for, $\beta_{0},\beta_{1},\beta_{2,}\mathrm{an}d\;\beta_{3}$ yields,

$$\begin{array}{c} \beta_{0}=-1.357\\ \beta_{1}=0.343\\ \beta_{2}=0.040\\ \beta_{3}=0.001 \end{array}$$

From Eq. (3), $K=10^{-1.357}$

Therefore, K=0.043

Knowing that, x=0.343,y=0.040,z=0.001

Finally, the mathematical model for predicting the tool wear shown in Table 5 is:

(7) $${\mathrm{Tw}}_{\max}=0.043.{V.}^{0.343}{f.}^{0.040}r^{0.001}$$

In order to determined percentage deviation shown in Table 5 in column 7 the mathematical model developed from experimental results, was used to predicted the tool wear, the predicted values and the experimental values were used as shown in Eq. (8) [10]

(8) $$\Phi_{i}=\left(\frac{{T_{w}}_{\left(p\right)\;-\;}{T_{w}}_{\left(e\right)}}{{T_{w}}_{\left(e\right)}}\right)\;\times \;100$$

where φ_i_: percentage deviation of single sample data, ${T_{w}}_{\left(e\right)}$: the experimental values of the tool wear, ${T_{w}}_{\left(p\right)}$_:_ predicted tool wear gotten from the mathematical model.

Similarly, the average percentage deviation ${\bar{\Phi }}_{i}$ is stated as [10];

(9) $${\bar{\Phi }}_{i}=\frac{\sum_{i=1}^{n}\Phi_{i}}{n}$$

where the average percentage deviation of all sample data and *n* is the number of experimental data. For the data

$${\bar{\Phi }}_{i}=\left[100-\left[\frac{16.096}{27}\right]\right]=99.4\%$$

The result of average percentage deviation means that the mathematical models could predict tool wear with 99.4% accuracy.

**Fig. 1:**
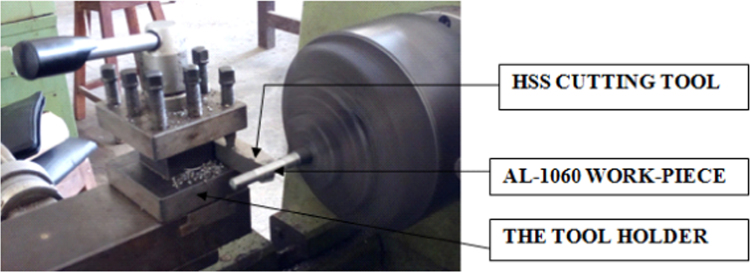
Experimental setup during the turning operation.

**Fig. 2:**
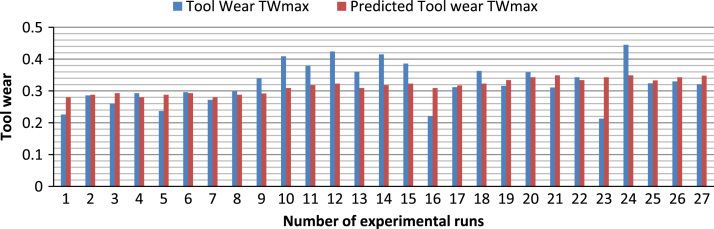
Comparison between experimental data and predicted data of tool wear (*TW_MAX_*).

**Fig. 3:**
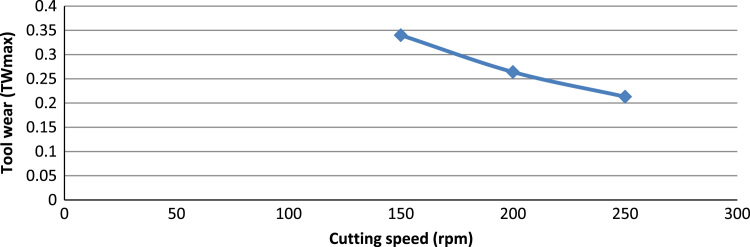
The effect of the cutting speed on the tool wear.

**Fig. 4:**
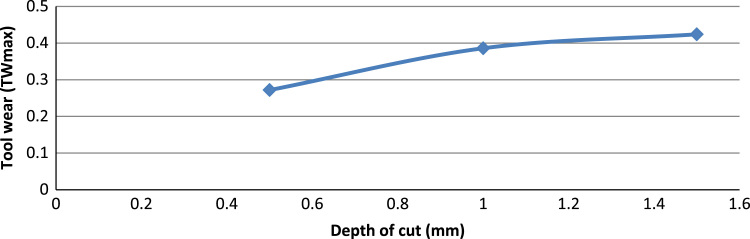
The effect of the depth of cut on the tool wear.

**Fig. 5:**
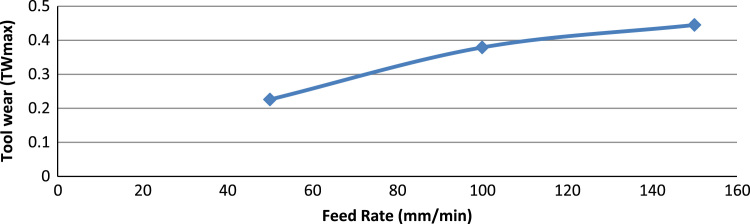
The effect of feed rate on the tool wear.

Fig. 1 shows the experimental setup of the turning operations. Table 1 shows the 6061-aluminium alloy chemical composition. Table 2, Table 3 show physical, mechanical properties of the work-piece and the influencing factors with their levels. The experimental results and the actual value, predicted value and percentage deviation of tool wear (*Tw_max_*) are shown in Table 4. Fig. 2, Fig. 3, Fig. 4, Fig. 5 show the comparison between the experimental data and the predicted data and the effects of the cutting parameters on the tool wear.

**Table 1:** Chemical composition of 1060 aluminium alloy.

Aluminum (Al)	99.6%
Iron (Fe)	0.35%
Silicon (Si)	0.24%
Copper (Cu)	0.05%
Vanadium (V)	0.04%
Zinc (Zn)	0.05%
Magnesium (Mg)	0.02%
Manganese (Mn)	0.03%
Titanium (Ti)	0.02%

**Table 2:** Physical properties of 1060 aluminium alloy.

Properties	Units
Density	2700 Kg/m^3^
Melting onset (Solidus)	646 °C
Tensile strength: Ultimate	70–130 MPa
Yield strength (Proof)	18–94 MPa
Elastic modulus	69 GPa

**Table 3:** The high speed steel composition.

Tungsten	18%
Chromium	4%
Vanadium	1%
Carbon	0.7%
Iron	Remained

**Table 4:** Process parameters and their levels used for the experiments.

Factors for machining	Levels and values		
	−1	0	+1
Cutting speed, *v* (m/min)	150	200	250
Feed, *f* (mm/min)	50	100	150
Depth of cut, *d* (mm)	0.5	1	1.5

**Table 5:** Experimental results and the comparison between experimental data and predicted data of tool wear (*TW_MAX_*).

Exp.nos.	Cutting speed(rpm)	Feed rate(mm/min)	Radial depth of cut(mm)	Tool wear	Predicted tool wear	Percentage deviation
				*TW_max_*	*TW_max_*	
1	150	50	1.5	0.226	0.28	23.893
2	150	100	1.5	0.286	0.288	0.699
3	150	150	1.5	0.26	0.293	12.692
4	150	50	1	0.293	0.28	−4.436
5	150	100	1	0.237	0.288	21.518
6	150	150	1	0.296	0.293	−1.013
7	150	50	0.5	0.272	0.28	2.941
8	150	100	0.5	0.299	0.288	−3.678
9	150	150	0.5	0.340	0.292	−14.117
10	200	50	1.5	0.409	0.309	−24.449
11	200	100	1.5	0.379	0.318	−16.094
12	200	150	1.5	0.424	0.323	−23.820
13	200	50	1	0.36	0.309	−14.166
14	200	100	1	0.415	0.318	−23.373
15	200	150	1	0.386	0.323	−16.321
16	200	50	0.5	0.221	0.309	39.819
17	200	100	0.5	0.312	0.317	1.602
18	200	150	0.5	0.363	0.323	−11.019
19	250	50	1.5	0.316	0.334	5.696
20	250	100	1.5	0.359	0.343	−4.456
21	250	150	1.5	0.311	0.349	12.218
22	250	50	1	0.343	0.334	-2.623
23	250	100	1	0.213	0.343	61.032
24	250	150	1	0.445	0.349	−21.573
25	250	50	0.5	0.324	0.333	2.777
26	250	100	0.5	0.33	0.343	3.939
27	250	150	0.5	0.321	0.348	8.411
						16.096
